# Ultrasonic‐Assisted Photocatalytic Conversion of Air to Nitric Acid Under Ambient Conditions

**DOI:** 10.1002/advs.202517167

**Published:** 2026-01-27

**Authors:** Shiming Chen, Hanlan Hong, Yunchuan Tu, Chao Zhen, Rui Zheng, Yuxi Yao, Junxiong Chen, Tianlin Wang, Xiumei Zhan, Fanhe Kong, Huazhe Yang, Gang Liu

**Affiliations:** ^1^ School of Intelligent Medicine China Medical University Shenyang China; ^2^ School of Chemistry and Chemical Engineering Chongqing University Chongqing China; ^3^ College of Land and Environment Shenyang Agricultural University Shenyang China; ^4^ Shenyang National Laboratory for Materials Science Institute of Metal Research, Chinese Academy of Sciences Shenyang China; ^5^ School of Materials Science and Engineering University of Science and Technology of China Shenyang China

**Keywords:** nitrogen activation, photocatalysis, sustainable catalysis, ultrasonic‐assisted reaction

## Abstract

The production of nitric acid (HNO_3_) holds significant importance in the chemical industry, and achieving direct air‐to‐HNO_3_ conversion with high efficiency and stability under mild conditions remains promising but challenging. Here, we demonstrate an ultrasound‐assisted photocatalytic method in which the generated hydroxyl (·OH) and superoxide (·O_2_^−^) radicals drive the direct synthesis of HNO_3_ from air under ambient conditions. The synthesis rate of HNO_3_ is up to 8594 µmol/h/g_cat_, over 200 times higher than that of previous photocatalytic methods. Using multiple ultrasound sources causes an additional eight times rate enhancement with nearly 100% selectivity maintained over 700 h. The resulting product exhibits comparable fertilization efficacy to conventional fertilizers. By harnessing the UV portion of sunlight that plants do not use, this approach paves the way for full‑spectrum solar energy utilization in sustainable agriculture.

## Graphical abstract

Ultrasound‐assisted photocatalytic air oxidation to nitrate enhances soil nitrogen fertilizer dynamics, thereby enabling improved energy conversion efficiency and sustainable agriculture.

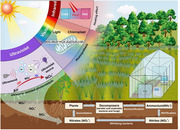

## 1 Introduction

Nitric acid (HNO_3_) is a fundamental chemical in various industrial applications, including the production of fertilizers, explosives, and precursors for nylon synthesis. Its industrial synthesis is primarily achieved via the Ostwald process, which involves ammonia oxidation with the help of Pt‐Rh catalysts at high temperatures of approximately 900°C [1].

$$\begin{array}{ccc} & & 2NH_{3}\left(g\right)+4O_{2}\left(g\right)\to 2H_{2}O\left(l\right)+2\mathrm{HN}O_{3}\left(\mathrm{aq}\right)\Delta H\\ & & \;=-894.66\;\mathrm{kJ}/\mathrm{mol} \end{array}$$

In this process, the feed gas ammonia is industrially synthesized from nitrogen (N_2_) and hydrogen (H_2_) via the Haber‐Bosch method, which is also an energy‐intensive process typically operating at high‐temperatures (400–500°C) and pressures (20–50 mPa), in addition to the large consumption of expensive H_2_ [2, 3]. This conventional two‐step production route thus involves significant energy input, considerable dissipation of thermal energy, and contributes heavily to global carbon emissions due to fossil‐derived hydrogen use [4].

In contrast, a direct oxidative conversion of N_2_–HNO_3_ represents a substantially more energy‐efficient and sustainable alternative, requiring significantly lower energy input:

$$N_{2}\;\left(g\right)+O_{2}\;\left(g\right)+H_{2}O\;\left(l\right)\;\to 2\mathrm{HN}O_{3}(\mathrm{aq})\;\Delta H=-128.97\;\mathrm{kJ}/\mathrm{mol}\;$$

Consequently, developing direct nitrogen oxidation methods is crucial for advancing sustainable and environmentally‐friendly nitrogen fixation technologies [3, 5, 6, 7, 8, 9], due to avoiding the alternative to the energy‐hungry Haber‐Bosch process, which is directly responsible for 1.5% of all global carbon dioxide emissions [10]. However, the exceptional stability of N_2_, characterized by its robust triple bond, poses a formidable challenge to achieving a high formation rate and selectivity of HNO_3_ sufficient for direct application as nitrogenous fertilizer. The previous findings show that the ultrasound treatment on water with dissolved air could produce nitrite and nitrate [11] and the microbubbles generated by ultrasound could produce HNO_3_ [12, 13]. But these processes show low productivity rate and yield of nitrate, which could not be directly used as nitrogenous fertilizer. Together with electrochemical NO_x_‐reduction technologies, our direct N_2_–NO_x_ oxidation establishes the complementary oxidative half of a unified, closed‐loop nitrogen cycle [14, 15, 16, 17, 18, 19]. Herein, by establishing an ultrasonic‐assisted photocatalytic process, we successfully achieved the direct conversion of atmospheric.

N_2_ and oxygen (O_2_) into nitric acid under mild conditions. This approach offers a sustainable and efficient pathway for producing nitrogen‐based fertilizers, contributing to enhanced agricultural productivity. The application of ultrasound significantly enhances the photocatalytic production of nitric acid, achieving a formation rate of 8594 µmol/h/g_cat._, which exceeds the highest previously reported rate for HNO_3_ production via photocatalysis over 200 times [20, 21, 22, 23, 24, 25, 26]. By employing multiply ultrasound source, the HNO_3_ productivity rate can be enhanced by a factor of eight compared with single ultrasound source, and this system shows the performance stability of 700 h with almost 100% HNO_3_ selectivity. The as‐produced solution directly used as nitrogenous fertilizer for cabbage culture shows the same performance as that of commercial fertilizers. Multiple in situ spectroscopic characterizations reveal that N_2_ is activated by the in situ‐generated OH to generate hyponitrous acid (H_2_N_2_O_2_) via nucleophilic addition, followed by further oxidization of H_2_N_2_O_2_–HNO_3_. Moreover, compared with the other ambient nitrogen activation process, such as electro‐conversion [15, 27, 28, 29, 30, 31, 32, 33, 34, 35, 36], this process shows higher production rate and stability. Integrated with renewable energy‐based electricity, this process provides a one‐step green route for the production of HNO_3_ by directly using the air resource and cheap device under mild conditions. This advancement facilitates the comprehensive exploitation of the entire solar spectrum, encompassing infrared (IR) light for heating environmental atmosphere, visible light for driving photosynthesis, and UV light for producing nitrogenous fertilizer, thereby providing the sustainable comprehensive utilization of solar energy‐for plant growth. This synergistic strategy enhances the efficiency of photocatalytic processes, leading to more sustainable and effective applications in environmental remediation and energy conversion.

## 2 Results and Discussion

### 2.1 Reaction Performance Assessment

**FIGURE 1:**
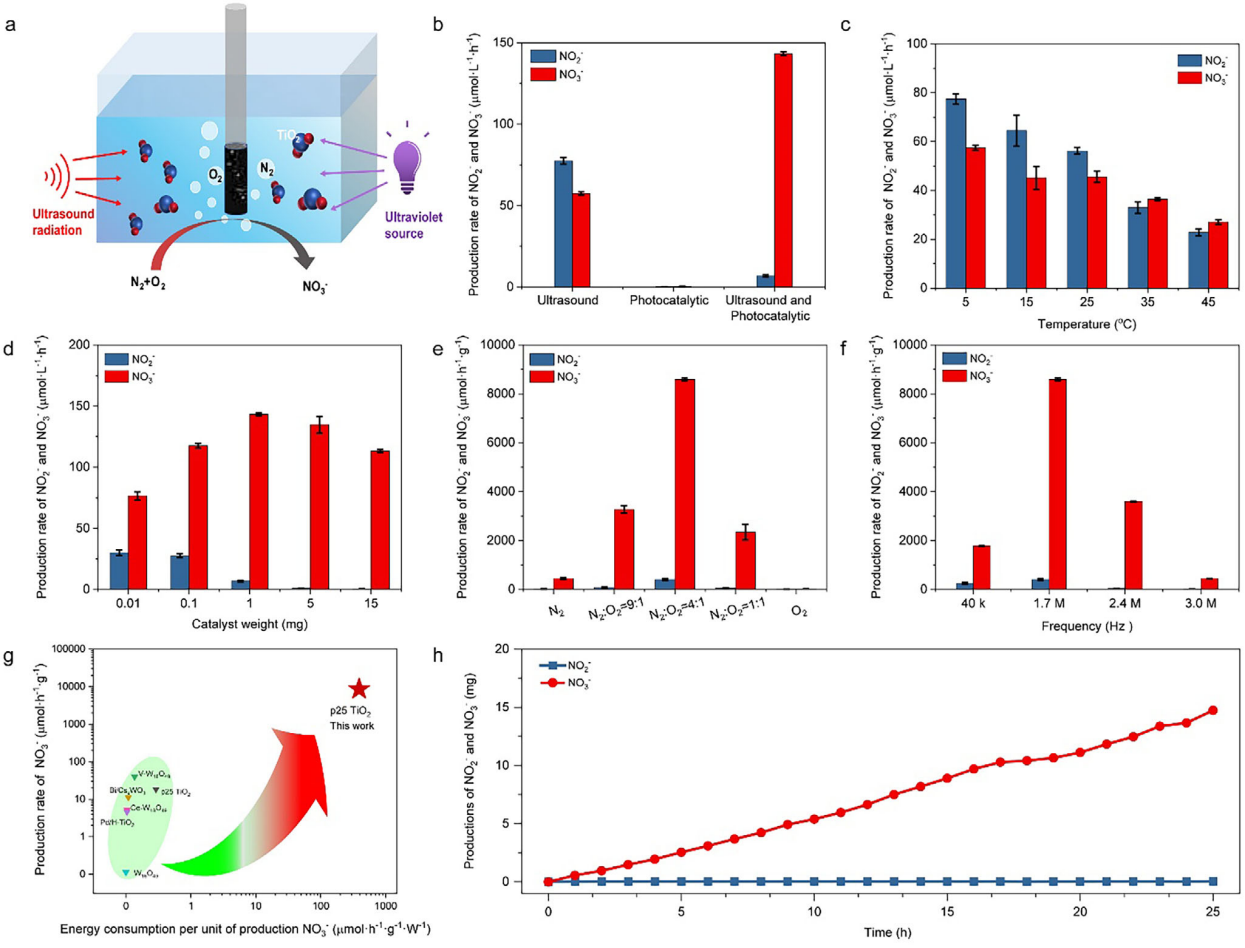
Catalytic performance testing for ultrasonic‐assisted photocatalytic conversion of air to HNO_3_. (a), Schematic diagram of home‐made reactor for ultrasonic‐assisted photocatalytic reaction. (b), HNO_3_ formation rates in the cases of ultrasound, photocatalytic and ultrasonic‐assisted photocatalytic process. (c), HNO_3_ formation rates at different temperatures under 1.7 mHz ultrasound irradiation. (d), HNO_3_ formation rate at different amount of photocatalysts. (e), HNO_3_ formation rate at different N_2_/O_2_ pressure ratios. (f), HNO_3_ formation rate at ultrasound irradiation of different frequences. (g), Comparison between the HNO_3_ formation rate and energy efficiency of ultrasonic‐assisted photocatalytic conversion of air to HNO_3_ and previously reported photocatalytic conversion of air or N_2_–HNO_3_. (h), Stability test of ultrasonic‐assisted photocatalytic process for 25 h. Each reaction was repeated three times to obtain the error bars. Typical reaction conditions: 60 mL water, P_O2_/P_N2_ =4/1, 31.5 W of 1.7 mHz ultrasound irradiation, 10 W LED 365 nm light source and 1 mg P25 photocatalysts.

The direct ultrasonic‐assisted photocatalytic conversion of atmospheric nitrogen to HNO_3_ was conducted in a home‐made reactor (Figure 1a). The final product was identified as nitric acid, as evidenced by the consistent correlation between the increase in proton concentration (pH decrease) and the rise in nitrate (NO_3_^−^) concentration (Figure S1). This integrated approach demonstrated superior performance in both the formation rate and selectivity of HNO_3_ when compared to individual photocatalytic or ultrasonic process (Figure 1b). Notably, the application of ultrasound alone resulted in the production of both NO_2_^−^ and NO_3_^−^ ions. In contrast, the standalone photocatalytic process yielded only minimal amounts of NO_2_^−^ and NO_3_^−^. However, when combining photocatalysis with ultrasound, the highest rates of HNO_3_ formation and selectivity were achieved. Within this system, ultrasound was applied to the reactor at various temperatures, revealing the temperature‐dependent formation rates of nitrite and nitrate (NO_2_^−^ and NO_3_^−^ ions decreased markedly as the temperature increased from 5 to 45°C (Figure 1c). This phenomenon can be attributed to the higher impact of reactant concentration than temperature during this·OH‐induced process, considering the high reactivity of·OH radicals. Notably, the production of·OH radicals by ultrasound is favored at low temperatures, while higher temperatures may lead to a decrease in free radical production [37]. Subsequent experiments investigated the influence of varying amounts of P25 photocatalyst on the reaction. The production rate of HNO_3_ peaked at 1 mg of P25 and declined with increasing catalyst amounts (Figure 1d). Notably, when the P25 amount exceeded 5 mg, a 100% selectivity for HNO_3_ was achieved. To further elucidate the nitrogen source for HNO_3_ production, mixtures of N_2_ and O_2_ with different partial pressure ratios (P_N2_/P_O2_) were utilized as the feed gas in the ultrasound‐assisted photocatalytic process. Control experiments using pure O_2_ as the feed gas revealed negligible HNO_3_ formation, indicating the absence of nitrogenous contaminants in the catalyst, solutions, or reactor (Figure 1e). In contrast, substantial HNO_3_ production occurred when N_2_/O_2_ mixtures were introduced, with the highest production rate observed at a P_N2_:P_O2_ ratio of 4:1. These findings confirm that the HNO_3_ is derived from N_2_. As depicted in Figure 1f, the maximum HNO_3_ formation rate of 8594 µmol h^−^^1^ g^−^^1^ was achieved at an ultrasound frequency of 1.7 mHz with an energy input power of 31.5 W, surpassing the previously reported highest photocatalytic HNO_3_ production rate by over 200 times. Considering the energy consumption of both ultrasound and light sources, an HNO_3_ formation rate per energy input of 207.9 µmol h^−^^1^ g^−^^1^ W^−^^1^ was attained, which is 447 times higher than the highest rate reported for photocatalytic processes (Figure 1g) [24]. Furthermore, a 25 h stability test demonstrated negligible degradation in both the productivity and selectivity of HNO_3_, indicating the consistent performance of this system (Figure 1h).

### 2.2 Reaction Mechanism Characterizations

**FIGURE 2:**
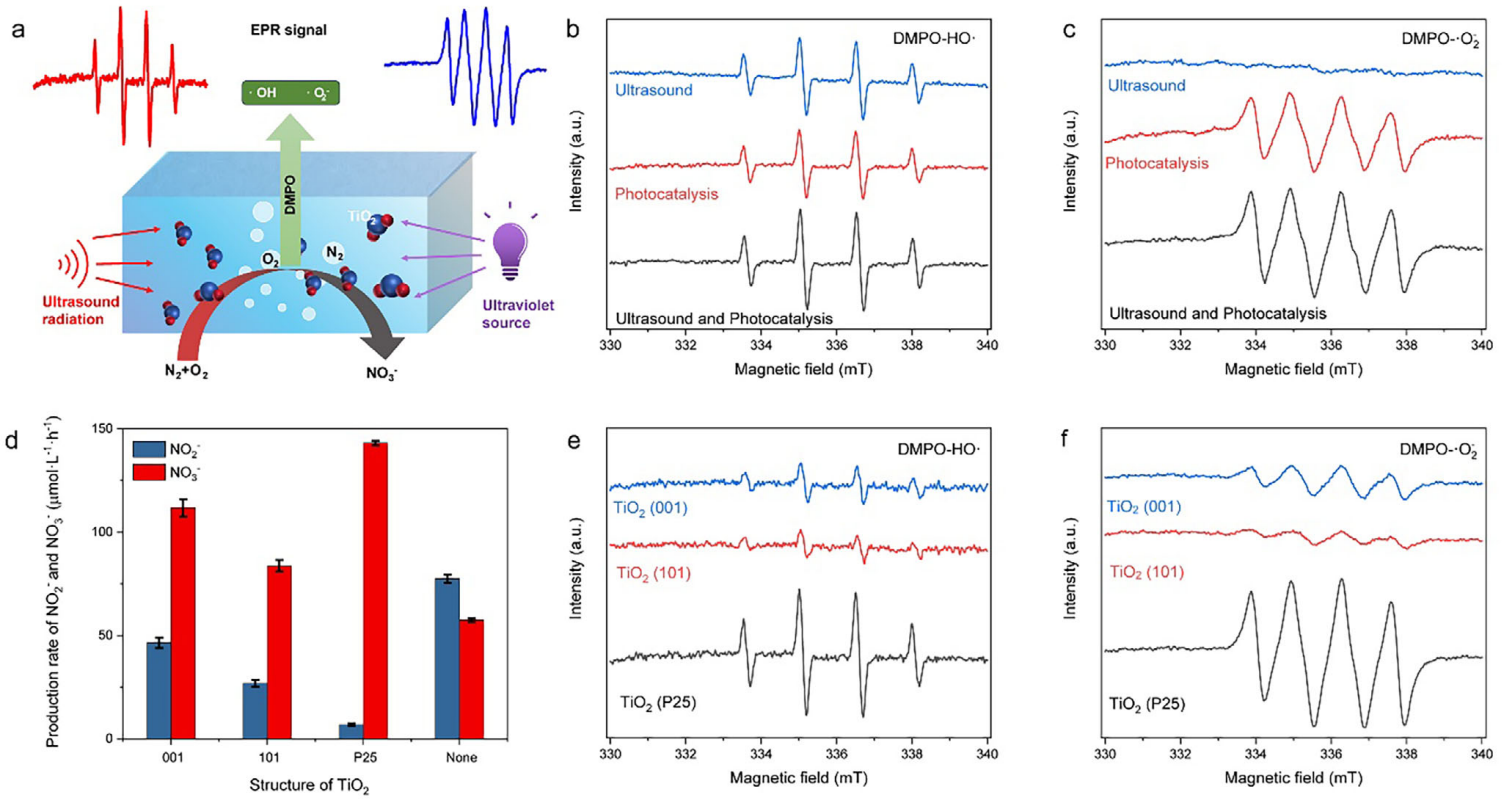
EPR study of oxygen‐containing transition states for ultrasonic‐assisted photocatalytic conversion of air to HNO_3_. (a), Schematic diagram of EPR detecting process of ultrasonic‐assisted photocatalytic process. (b), DMPO spin‐trapping EPR spectra of produced hydroxyl radicals in 40 mL solution under ultrasound, photocatalysis and ultrasound‐assisted photocatalysis. (c), DMPO spin‐trapping EPR spectra of produced superoxide radicals in 40 mL solution under ultrasound, photocatalysis and ultrasound‐assisted photocatalysis. (d), HNO_3_ formation rates with TiO_2_ of different crystal structures. (e) DMPO spin‐trapping EPR spectra of produced hydroxyl radicals with different photocatalysts in 1 mL solution under photocatalysis. (f), DMPO spin‐trapping EPR spectra of produced superoxide radicals with different photocatalysts in 1 mL solution under photocatalysis. Typical reaction conditions: 60 mL water, P_O2_/P_N2_ =4/1, 31.5 W of 1.7 mHz ultrasound irradiation, 10 W LED 365 nm light source and 1 mg photocatalysts.

To elucidate the reaction mechanism underlying the ultrasonic‐assisted photocatalytic conversion of air to HNO_3_, a series of in situ spectroscopic characterizations coupled with mass spectrometric analyses were performed to identify key reaction intermediates. In order to further unravel the synergistic interplay between ultrasonication and photocatalysis, electron paramagnetic resonance (EPR) spectroscopy was employed to detect and quantify the free radicals generated in the reaction system. As reactive oxygen species (ROS) are known to play a pivotal role in nitrogen activation such as hydroxyl radicals (·OH) and superoxide anions (·O_2_^−^), 5,5‐dimethyl‐1‐pyrroline N‐oxide (DMPO) was employed as a spin‐trapping agent. For the detection of·OH radicals, DMPO was utilized in aqueous solutions, forming DMPO‐·OH adducts. Conversely, to detect·O_2_^−^ radicals, DMPO was used in methanol solutions, facilitating the formation of DMPO‐O_2_^−^ adducts [38, 39, 40]. EPR spectroscopy was utilized to detect DMPO‐·OH adduct signals under ultrasound, photocatalysis, and combined ultrasound‐assisted photocatalysis conditions (Figure 2a,b). Notably, the signal intensity observed in the combined ultrasound‐assisted photocatalytic system was significantly higher than that detected under individual ultrasound or photocatalysis conditions, indicating a synergistic enhancement in·OH generation when both processes are simultaneously applied. Furthermore, EPR spectroscopy revealed that DMPO‐O_2_^−^ adduct signals were undetectable under ultrasound alone but present during the photocatalytic process (Figure 2c). Moreover, the combined ultrasound‐assisted photocatalytic process exhibited higher DMPO‐O_2_^−^ signal intensities compared to individual photocatalysis. This finding suggests that·O_2_^−^ play a crucial role in facilitating the oxidation of NO_2_^−^ to nitrate NO_3_^−^ ions. Collectively, these results highlight the enhanced efficiency and effectiveness of the combined ultrasound‐assisted photocatalytic approach in radical generation and oxidative transformations. To further substantiate the essential roles of the reactive species, we combined salicylic‐acid probing with methanol‐quenching experiments under typical reaction conditions. The salicylic‐acid assay reveals that photocatalysis alone produces only trace ·OH—insufficient to induce N_2_ activation—whereas the ultrasound‐assisted photocatalytic system exhibits a pronounced decrease in the 295 nm band, indicative of a substantially elevated·OH flux(Figure S7). In parallel, the complete suppression of NO_3_^−^ and NO_2_^−^ upon addition of methanol, a selective·OH scavenger with negligible reactivity toward O_2_^−^ (Figure S8), confirms that·OH is the indispensable initiating species for N_2_ activation, while·O_2_^−^ alone cannot sustain the oxidative pathway leading to HNO_3_. Building upon these findings, we have developed an ultrasound‐assisted photocatalytic system for air conversion under mild conditions. The total nitrogen fixation achieved under the combined ultrasound–photocatalytic conditions is approximately 15% higher than that obtained with ultrasound alone, further demonstrating the synergistic enhancement arising from coupling photogenerated charge carriers with acoustic cavitation. In addition, three samples of titanium dioxide (TiO_2_) photocatalysts with distinct features, specifically anatase TiO_2_ with dominant (001) facets exposed (denoted as TiO_2_ (001)), anatase TiO_2_ with dominant (101) facets exposed (denoted as TiO_2_ (101)), and P25 with the mixture of anatase and rutile phases (denoted as TiO_2_ (P25)), were evaluated. Among these, the P25‐type TiO_2_ exhibited the highest formation rate and selectivity for HNO_3_ production (Figure 2d). To elucidate the mechanisms underlying this enhanced performance, EPR measurements detected DMPO‐OH and DMPO‐O_2_^−^ adduct signals for each photocatalyst (Figure 2e,f), with P25 showing significantly higher signal intensities than TiO_2_ (001) and TiO_2_ (101). This superior photocatalytic activity of P25 is attributed to the presence of a metastable intermediate structure at the anatase/rutile interface, which facilitates more efficient charge separation, thereby enhancing photocatalytic efficiency relative to single‐phase TiO_2_ [41].

**FIGURE 3:**
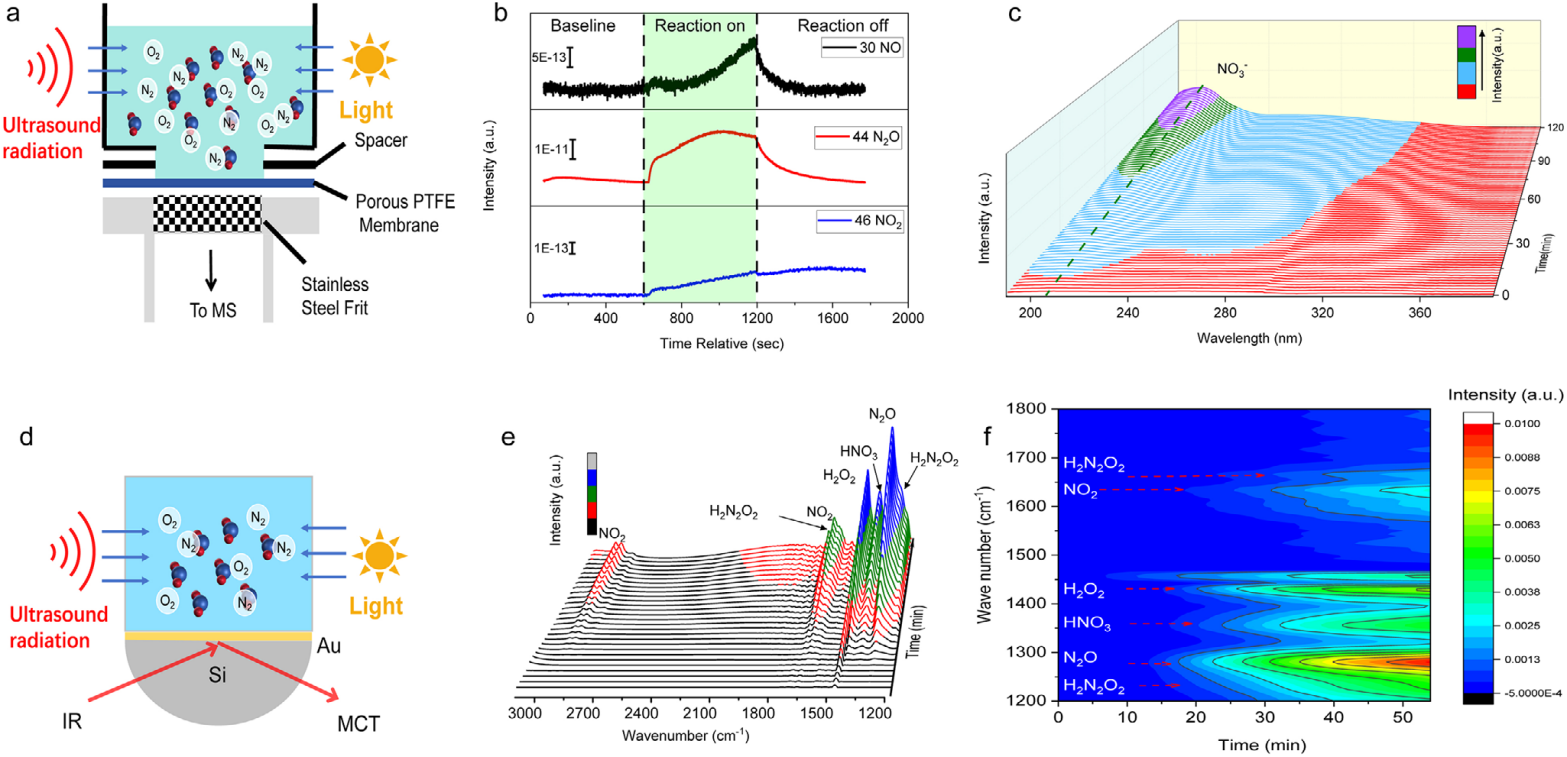
Investigation of the reaction intermediates in the ultrasonic‐assisted photocatalytic conversion of air to HNO_3_. (a), Schematic illustration of studying the ultrasonic‐assisted photocatalytic process with in situ mass spectrum. (b), Gas products detected by in situ mass spectrum. (c), In situ UV–vis absorbance characterization of the reaction process. (d), Schematic illustration of studying the ultrasonic‐assisted photocatalytic process with in situ surface‐enhanced infrared absorption spectroscopy (SEIRAS). (e), In situ SEIRAS characterization of the reaction process. (f), Contour plot of the in situ SEIRAS spectra for the peaks of intermediates. Typical reaction conditions: 60 mL water, P_O2_/P_N2_ =4/1, 31.5 W of 1.7 mHz ultrasound irradiation, 10 W LED 365 nm light source and 1 mg P25 photocatalysts.

In situ mass spectrometry detected gaseous intermediates, specifically nitrogen monoxide (NO), nitrous oxide (N_2_O), and nitrogen dioxide (NO_2_), corresponding to mass‐to‐charge ratios (m/z) of 30, 44, and 46, respectively (Figure 3a). The intensities of these signals increased during the reaction process (Figure 3b), confirming that the produced HNO_3_ originates from molecular nitrogen (N_2_). Furthermore, in situ UV–vis spectrophotometric analysis of the reaction process revealed an increasing absorption peak around 206 nm (Figure 3c), which corresponds to the characteristic absorption of NO_3_^−^. In situ surface‐enhanced infrared absorption spectroscopy (SEIRAS) analysis of the reaction process revealed the formation of the H_2_N_2_O_2_ intermediate, as evidenced by the gradual increase of two absorption bands at 1230 and 1670 cm^−^^1^ (Figure 3d,e), corresponding to the N─N and N─O stretching vibrations of H_2_N_2_O_2_, respectively. In situ SEIRAS analysis during the mid‐stage of the reaction process identified NO_2_ as an intermediate, evidenced by the emergence of absorption peaks at 1630 and 2900 cm^−^^1^ (Figure 3e). The 1630 cm^−^^1^ peak overlaps with the 1670 cm^−^^1^ peak of H_2_N_2_O_2_, resulting in an intensified signal around 1650 cm^−^^1^. Additionally, N_2_O was detected as a byproduct, indicated by an absorption peak at 1278 cm^−^^1^, likely originating from the decomposition of H_2_N_2_O_2_. The formation of hydrogen peroxide (H_2_O_2_) was confirmed by the appearance of a peak at 1428 cm^−^^1^. The final product, HNO_3_ was observed through the emergence of an absorption peak at 1359 cm^−^^1^ at the conclusion of the reaction process. Building upon the aforementioned investigations and characterizations, we propose a reaction pathway for the ultrasonic‐assisted photocatalytic conversion of atmospheric N_2_–HNO_3_. Initially, molecular O_2_ present in air undergoes heterogeneous reduction to H_2_O_2_, which is subsequently converted to·OH through both ultrasound and photocatalytic process. These·OH radicals then activate N_2_, leading to its oxidation into NO_2_^−^ via H_2_N_2_O_2_ as a key intermediate (Figure 3f). And the NO_2_^−^ was further oxidated to HNO_3_ by·O_2_^−^ generated photocatalytic process. This proposed mechanism aligns with our previous work [36] and is further corroborated by the findings of R. N. Zare et al. [12] who identified H_2_N_2_O_2_, NO, NO_2_, and NO_2_^−^ as intermediates preceding the formation of HNO_3_ as the final product.

$$\begin{array}{ccc} & & N_{2}\overset{\cdot OH}{\to }H_{2}N_{2}O_{2}\overset{\cdot OH}{\to }2NO+2H_{2}O\overset{\cdot OH}{\to }2HNO_{2}+2H_{2}O\overset{\cdot O_{2}^{-}}{\to }2HNO_{3}\\ & & \;+\;4H_{2}O \end{array}$$

### 2.3 Scale Up Experiments and Their Applications in Plant Cultivation

**FIGURE 4:**
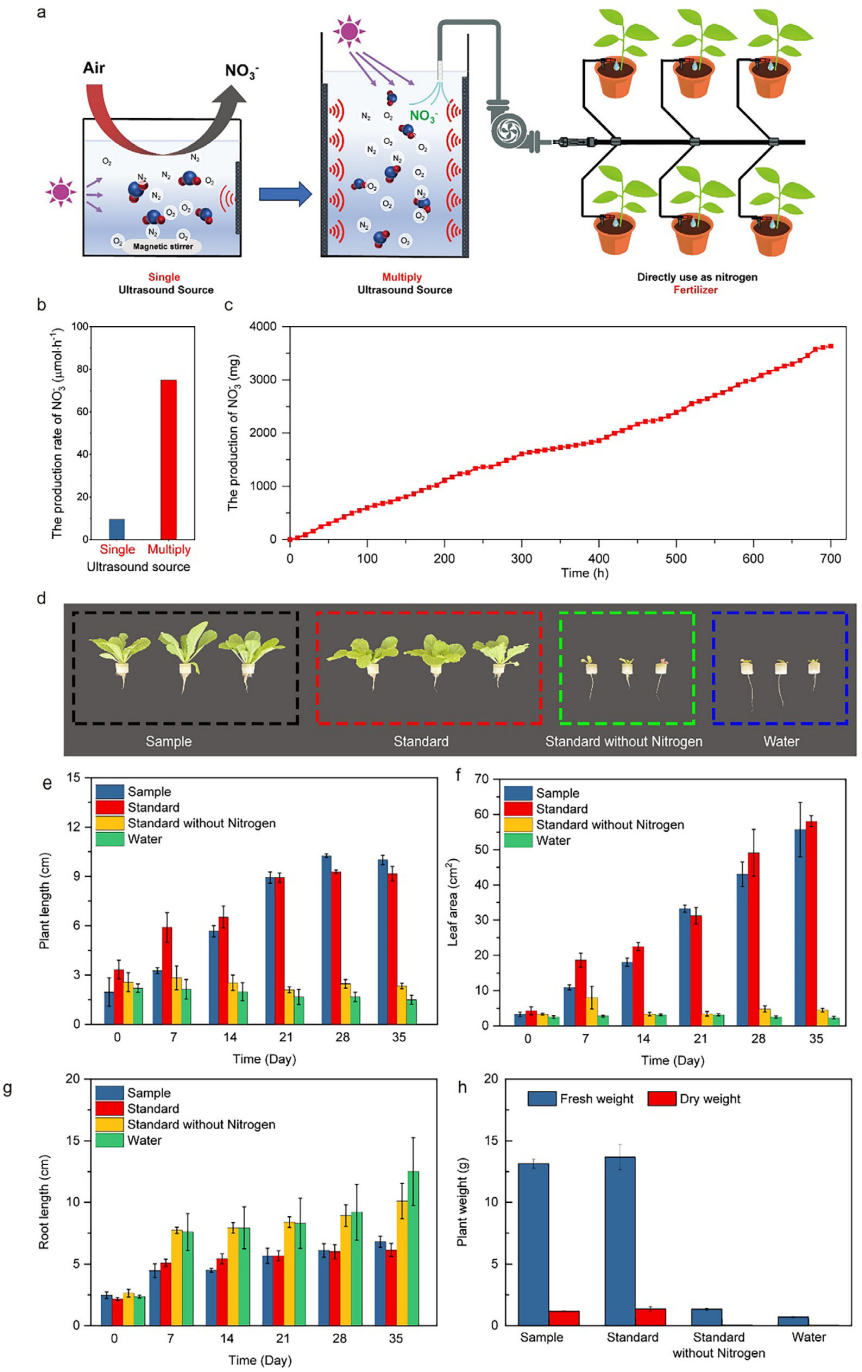
Scaling Up Ultrasonic‐Assisted Photocatalytic Processes and Their Application in Plant Cultivation. (a), Schematic representation of the scaled‐up ultrasonic‐assisted photocatalytic system designed for plant cultivation applications. (b), Comparison of nitric acid (HNO_3_) formation rates utilizing single vs. multiple ultrasound sources. (c), Stability assessment of the ultrasonic‐assisted photocatalytic process over a duration of 700 h. (d), Photographs of pakchoi cabbage after 35 days of cultivation under four different conditions: 1, Nitric acid‐based nutrient solution produced by the system. 2, Standard Hoagland nutrient solution. 3, Hoagland nutrient solution devoid of a nitrogen source. 4, Pure water. (e), Temporal statistical analysis of plant height. (f), Temporal statistical analysis of leaf area. (g), Temporal statistical analysis of root length. (h), Temporal statistical analysis of plant biomass (fresh and dry weight). Each data point represents the mean values of three replicates, with error bars indicating standard deviations.

To produce sufficient HNO_3_ for evaluating its efficacy as a fertilizer in plant cultivation, a multi‐ultrasound source photocatalytic reaction system was designed (Figure 4a). In the scaled reactor, uniform cavitation and photonic fields were maintained by employing multiple ultrasound transducers together with strip‐type UV lamps immersed in the solution, thereby ensuring homogeneous radical generation and underpinning the eightfold enhancement in HNO_3_ productivity.Optimizing the distribution of ultrasound and light sources resulted in the HNO_3_ production rate of up to 75 µmol/h, an eightfold increase compared to a single ultrasound source (Figure 4b). Stability tests over 700 h demonstrated consistent HNO_3_ production rates (Figure 4c). The nitric acid produced by our system was then used for the cultivation of pakchoi cabbage in a daylight greenhouse with a temperature range of 25–35°C. Seedlings were initially grown in trays and transplanted into 100 mL black cultivation bottles upon reaching 3–4 leaves. Then the pakchoi cabbage were cultured in: 1, Nitric acid‐based nutrient solution produced by the system; 2, Hoagland nutrient solution; 3, Hoagland nutrient solution without nitrogen; 4, pure water. All of the nutrient solutions, except for the pure water group, had identical nutrient compositions, with the only difference being the nitrogen source, aiming to evaluate the effect of the novel nitrogen fertilizer. Each treatment was replicated three times. During the initial two weeks, plants were administered nutrient solutions at half concentration; subsequently, full‐concentration solutions were applied for the remaining three weeks. Nutrient solutions were refreshed weekly throughout the five‐week cultivation period. As shown in Figure 4d, plants treated with the system‐produced nitric acid‐based fertilizer, as well as those supplied with Hoagland solution, demonstrated vigorous and sustained growth throughout the cultivation period. In contrast, plants grown in nitrogen‐free media or plain water showed poor development and failed to thrive (Figure 4d). Plant height and leaf area increased over time in the HNO_3_ and Hoagland groups, while minimal growth occurred in the nitrogen‐free and water groups (Figure 4e,f). Interestingly, root growth was more pronounced in the nitrogen‐free nutrient solution compared to the other groups (Figure 4g). Comparisons of fresh and dry weights revealed that yields from the HNO_3_ produced by our system were comparable to those from the standard nutrient solution (Figure 4h). In summary, nitric acid produced via ultrasound‐assisted photocatalysis effectively meets the nitrogen fertilizer requirements for plant growth. This process, utilizing renewable energy under mild conditions, offers a sustainable pathway for nitrogen fixation.

## 3 Conclusion

In this study, we present an ultrasonic‐assisted photocatalytic route for the direct conversion of air to HNO_3_ with a remarkable enhancement on productive efficiency and stability at ambient conditions. The application of ultrasound significantly enhances the photocatalytic production of nitric acid (HNO_3_), achieving a formation rate of 8594 µmol/h/g, which exceeds the previously reported highest rate for HNO_3_ production via photocatalysis over 200 times. Multiple spectroscopic characterizations reveal that N_2_ is efficiently activated by ·OH generated through the ultrasound and photocatalytic process, leading to its oxidation into NO_2_^−^ via hyponitrous acid (H_2_N_2_O_2_) as a key intermediate. The generated NO_2_^−^ is further oxidized to HNO_3_ by superoxide radicals (·O_2_^−^) produced during the photocatalytic process. This mechanistic pathway highlights the synergistic effects of ultrasound and photocatalysis in nitrogen activation. The nitrogen‐contained fertilizer produced has comparable fertility on pakchoi cabbage compared to the traditional Hoagland nutrient solution, indicating that our approach can provide a valuable feedstock on nitrogen fixation. This synergistic strategy enhances the efficiency of photocatalytic processes, leading to more sustainable and effective applications in environmental remediation and energy conversion.

## Author Contributions

G.L. conceived the project. S.M.C. and H.L.H. carried out the experiments and the manuscript preparation. G.L. and H.Z.Y. supervised the experiment and manuscript revision. Y.C.T. performed DEMS and in‐situ IR. F.H.K. and X.M.Z. performed the cultivation of the plants. C.Z., R.Z., Y.X.Y., J.X.C. and T.L.W. assisted with experiments and data analysis. All authors contributed to scientific discussion of the manuscript. S.M.C., H.Z.Y., and G.L. wrote the paper.

## Funding

National Natural Science Foundation of China 52425201 (GL); National Natural Science Foundation of China 22402229 (SMC); New Cornerstone Science Foundation through the XPLORER PRIZE (GL).

## Conflicts of Interest

The authors declare no conflicts of interest.

## Supporting information




**Supporting File**: advs73686‐sup‐0001‐SuppMat.docx.

## Data Availability

The data that support the findings of this study are available from the corresponding author upon reasonable request.
