# Supplementary material for: Ultrasonic‐Assisted Photocatalytic Conversion of Air to Nitric Acid Under Ambient Conditions
Source: Adv Sci (Weinh). 2026 Jan 27;13(16):e17167. doi: 10.1002/advs.202517167 (PMC13042811; doi:10.1002/advs.202517167)
Supplement: Supplementary file 1 — Supporting File: advs73686‐sup‐0001‐SuppMat.docx. [file ADVS-13-e17167-s001.docx]

Supporting information for

Ultrasonic-assisted photocatalytic conversion of air to nitric acid under ambient conditions

**This PDF file includes:**

Experimental Section

Figs. S1 to S13

Tables S1

**Experimental Section**

**Materials and chemicals**

All chemicals are analytical grade commercial materials without further purification. P25 titanium dioxide produced by the gas phase production process of Degussa was used as catalyst. Argon (99.99%), oxygen (99.995%), nitrogen (99.99%) and synthetic air (N_2_/O_2_ = 4/1) are supplied by Xulong Gas Co., LTD.

**Products detection and characterization instruments**

A UV-vis spectrophotometer (UV-1900, Shimadzu) with a homemade *in situ* cell were employed for intermediate products detection. Quantitative determination of the formed HNO_3_ were detected by Ion chromatography (SHINE TSINGTAO CIC-D100).

Detection of ·OH and ·O_2_^-^ during the reaction

EPR spectroscopic measurements were performed on a Bruker Magnettech ESR5000 spectrometer operating at the X-band frequency with a microwave frequency of 9.43 GHz, a microwave power of 100 mW and a modulation frequency of 100 kHz. The EPR characterizations of the **·**OH were conducted by using DMPO (2 mmol/L) as a capture agent in water solution and **·**O_2_^-^ were conducted by using DMPO (4 mmol/L) as a capture agent in menthol solution.

**Reaction conditions**

The reactor was a double-layer photocatalytic cell with a volume of 60 mL and a quartz light window of 2 cm in diameter. A 365 nm ultraviolet light lamp (10 W) was placed at one end of the light window, while the ultrasonic vibration plate was placed on the opposite side. 60 mL of ultra-pure water and the catalyst was employed introduced to the reactor. Simultaneously, 60 mL/min of gas was introduced into the reactor. The reaction was maintained for 60 minutes.

**Ultrasonic assisted photocatalytic process measurements with in situ** **SEIRAS**

The *in situ* SEIRAS was measured with attenuated total reflection (ATR) SEIRAS. The IR beam goes through the prism and reaches reaction solution which was pumped from the reactor. Before the experiment, the Au film was deposited on Si prism surface to have Surface Enhancement IR Spectra. All spectra were presented in absorbance, A = −log(R/R_0_), where R is the reflectance of the sample spectrum and R_0_ is the background spectrum (0 min). A BRUKER VERTEX 70v FTIR spectrometer was used for the SEIRAS measurements.

**Reaction rates calculation**

The yield of NO_3_^-^ was calculated through the following equation:

$$r_{{NO}_{3}^{-}}(\mu mol/h/g)=\frac{x\left( ppm \right)\cdot V\left( mL \right)}{m(g)\cdot t\left( h \right)\cdot Mr\left( g/mol \right)}$$

where:

$\mathbf{r}_{\mathbf{NO}_{\mathbf{3}}^{\mathbf{-}}}$ : NO_3_^-^ formation rate in$\boldsymbol{\mu mol/h/g}$;

**x (ppm)**: NO_3_^-^ concentration detected by IC in ppm **(*μg/mL*)**;

**V (mL)**: The volume of electrolyte in **milliliter**;

**m(g):** The weight of catalyst in **gram**;

$\mathbf{Mr(g/mol)}$: Molar mass of NO_3_^-^ in $\boldsymbol{g/mol}$.

The yield of NO_2_^-^ was calculated through the following equation:

$$r_{{NO}_{2}^{-}}(\mu mol/h/g)=\frac{x\left( ppm \right)\cdot V\left( mL \right)}{m(g)\cdot t\left( h \right)\cdot Mr\left( g/mol \right)}$$

where:

$\mathbf{r}_{\mathbf{NO}_{\mathbf{2}}^{\mathbf{-}}}$ : NO_2_^-^ formation rate in$\boldsymbol{\mu mol/h/g}$;

**x (ppm)**: NO_2_^-^ concentration detected by IC in ppm **(*μg/mL*)**;

**V (mL)**: The volume of electrolyte in **milliliter**;

**m(g):** The weight of catalyst in **gram**;

$\mathbf{Mr(g/mol)}$: Molar mass of NO_2_^-^ in $\boldsymbol{g/mol}$.

**Energy efficiency calculation**

The total **Energy efficiency** (EE) is calculated as:

$$\text{E}\text{E}=\frac{r_{\mathrm{HN}O_{3}}}{P_{\text{light}}+P_{\text{ultrasound}}}$$

where $r_{\mathrm{HN}O_{3}}$is the HNO₃ production rate (µmol h⁻¹ g⁻¹), and $P_{\text{light}}$and $P_{\text{ultrasound}}$are the electrical power inputs for the 365 nm LED (10 W) and the ultrasound transducer (31.5 W), respectively.

**Figure S1.** Time-dependent variation in proton concentration, as indicated by changes in pH during the reaction process.

a b

c

**Figure S2.** **NO_2_^-^ and NO_3_^-^ quantification with ion chromatography (IC).** (a) IC profiles of virous concentration of standard samples. (b) Calibration curve for quantification of NO_3_^-^. (c) Calibration curve for quantification of NO_2_^-^.

a b

c

**Figure S3.** **IC profiles of three independent reaction data with different ratio of N_2_ and O_2_**. Typical reaction conditions: 60 mL water, P_O2_/P_N2_ =4/1, 31.5 W of 1.7 MHz ultrasound irradiation, 10 W LED 365 nm light source and 1 mg P25 photocatalysts.

a b

c

**Figure S4**. **IC profiles of three independent reaction data with different** **ultrasound frequency**. Typical reaction conditions: 60 mL water, P_O2_/P_N2_ =4/1, 31.5 W of 1.7 MHz ultrasound irradiation, 10 W LED 365 nm light source and 1 mg P25 photocatalysts.

a b

c

**Figure S5**. **IC profiles of three independent reaction data with different** **temperature**. Typical reaction conditions: 60 mL water, P_O2_/P_N2_ =4/1, 31.5 W of 1.7 MHz ultrasound irradiation, 10 W LED 365 nm light source and 1 mg P25 photocatalysts.

a b

c

**Figure S6**. **IC profiles of three independent reaction data with different** **weight of catalyst.** Typical reaction conditions: 60 mL water, P_O2_/P_N2_ =4/1, 31.5 W of 1.7 MHz ultrasound irradiation, 10 W LED 365 nm light source and 1 mg P25 photocatalysts.

**Table S1. Comparison of the rates of direct oxidation of nitrogen to nitrogen oxides by photocatalysis in recent years.**

| Catalysts | Reaction type | Light source | Atmosphere | Rate (µmol g^−1^ h^−1^) |  |
| --- | --- | --- | --- | --- | --- |
| P25 TiO_2_ | Ultrasonic- assisted Photocatalysis | 10W LED  (365 nm) | Air | 8594.3 | *This work* |
| P25 TiO_2_ | Ultrasonic- assisted Photocatalysis | 300W Xenon lamp | Air | 5583.04 | *This work* |
| 001 TiO_2_ | Ultrasonic- assisted Photocatalysis | 10W LED  (365 nm) | Air | 6706.22 | *This work* |
| 001F TiO_2_ | Ultrasonic- assisted Photocatalysis | 10W LED  (365 nm) | Air | 6365.23 | *This work* |
| 101 TiO_2_ | Ultrasonic- assisted Photocatalysis | 10W LED  (365 nm) | Air | 5023.13 | *This work* |
| 101F TiO_2_ | Ultrasonic- assisted Photocatalysis | 10W LED  (365 nm) | Air | 6147.92 | *This work* |
| TiO_2_ | photocatalysis | 40W LED  (365 nm) | N_2_ | 18.5 | *Angewandte Chemie International Edition 61, e202211469 (2022)* |
| Pd/H-TiO_2_ | Photothermal-assisted Photocatalysis | 300W Xenon lamp | N_2_ | 4.58 | *Advanced Energy Materials 12,2103740 (2022)* |
| W_18_O_49_ | photocatalysis | 300W Xenon light | N_2_ | 0.057 | *Research (Washington, D.C.),3750314 (2020)* |
| Ce-W_18_O_49_ | photocatalysis | 300W Xenon light | N_2_ | 5.16 | *The Journal of Physical Chemistry Letters 12, 11295-11302 (2021)* |
| V-W_18_O_49_ | photocatalysis | 300W Xenon light | N_2_ | 39.85 | *Applied Catalysis B: Environmental,343,123539(2024)* |
| Bi/Cs_x_WO_3_ | photocatalysis | 300W Xenon light | N_2_ | 11.2 | *Angew Chem Int Ed Engl, 62(47) e202311911*  *(2023)* |

Figure S7. **Salicylic-acid probe spectra for evaluating ·OH generation.**

UV–vis spectra of salicylic acid under different reaction conditions. The decrease in the 295 nm absorption band reflects ·OH-induced oxidation of salicylic acid. Photocatalysis alone shows only minimal SA consumption—close to the background—indicating very limited ·OH production, whereas the ultrasound-assisted photocatalytic condition results in a pronounced decrease of the 295 nm peak, confirming significantly enhanced ·OH generation. Typical reaction conditions: 60 mL water, P_O2_/P_N2_ =4/1, 1.7 MHz ultrasound irradiation, 10 W LED 365 nm light source and 1 mg photocatalysts.


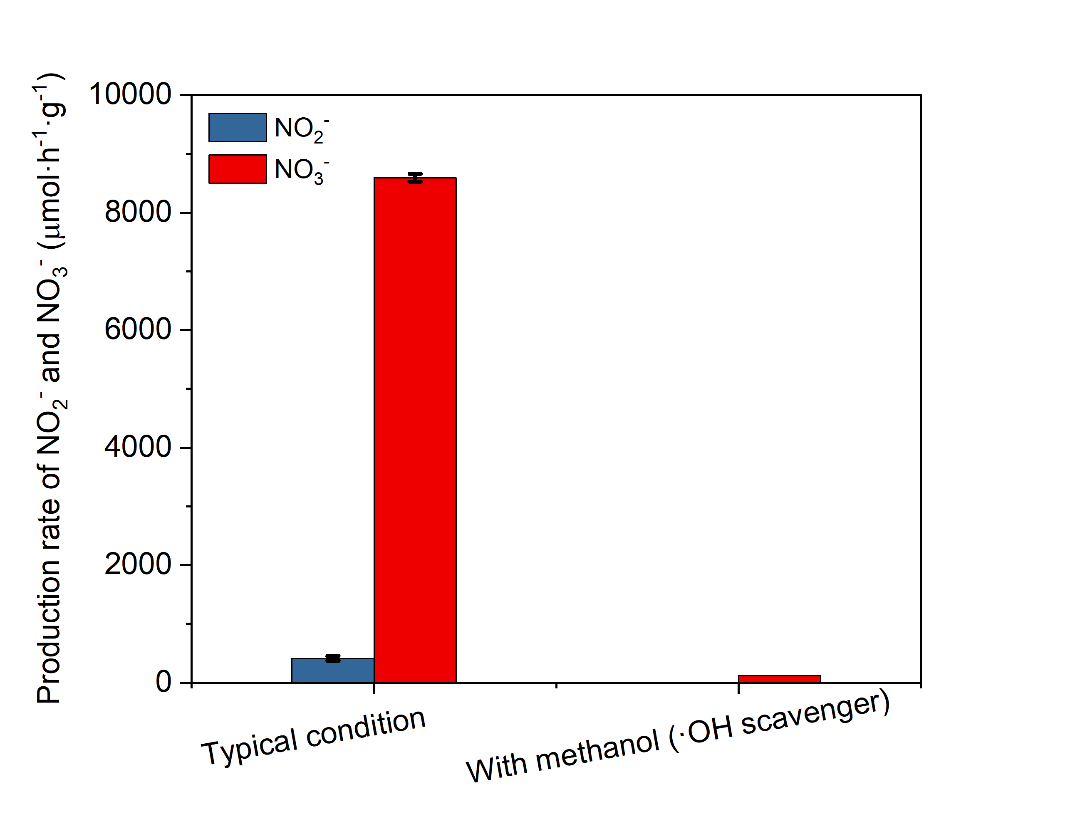
Figure S8. **Methanol-quenching experiment under typical ultrasound-assisted photocatalytic conditions.** Typical reaction conditions: 60 mL water, P_O2_/P_N2_ =4/1, 31.5 W of 1.7 MHz ultrasound irradiation, 10 W LED 365 nm light source and 1 mg photocatalysts.

**Figure S9.** **Catalytic performance testing for ultrasonic-assisted photocatalytic conversion of air to HNO_3_.** HNO_3_ formation rates in the cases of ultrasound, photocatalytic and ultrasonic-assisted photocatalytic process. Typical reaction conditions: 60 mL water, P_O2_/P_N2_ =4/1, 31.5 W of 1.7 MHz ultrasound irradiation, 10 W LED 365 nm light source and 1 mg photocatalysts.

**Figure S10. XRD patterns of pristine and used TiO₂ (P25) catalysts.** The identical diffraction features confirm the excellent structural stability of the material throughout the reaction.


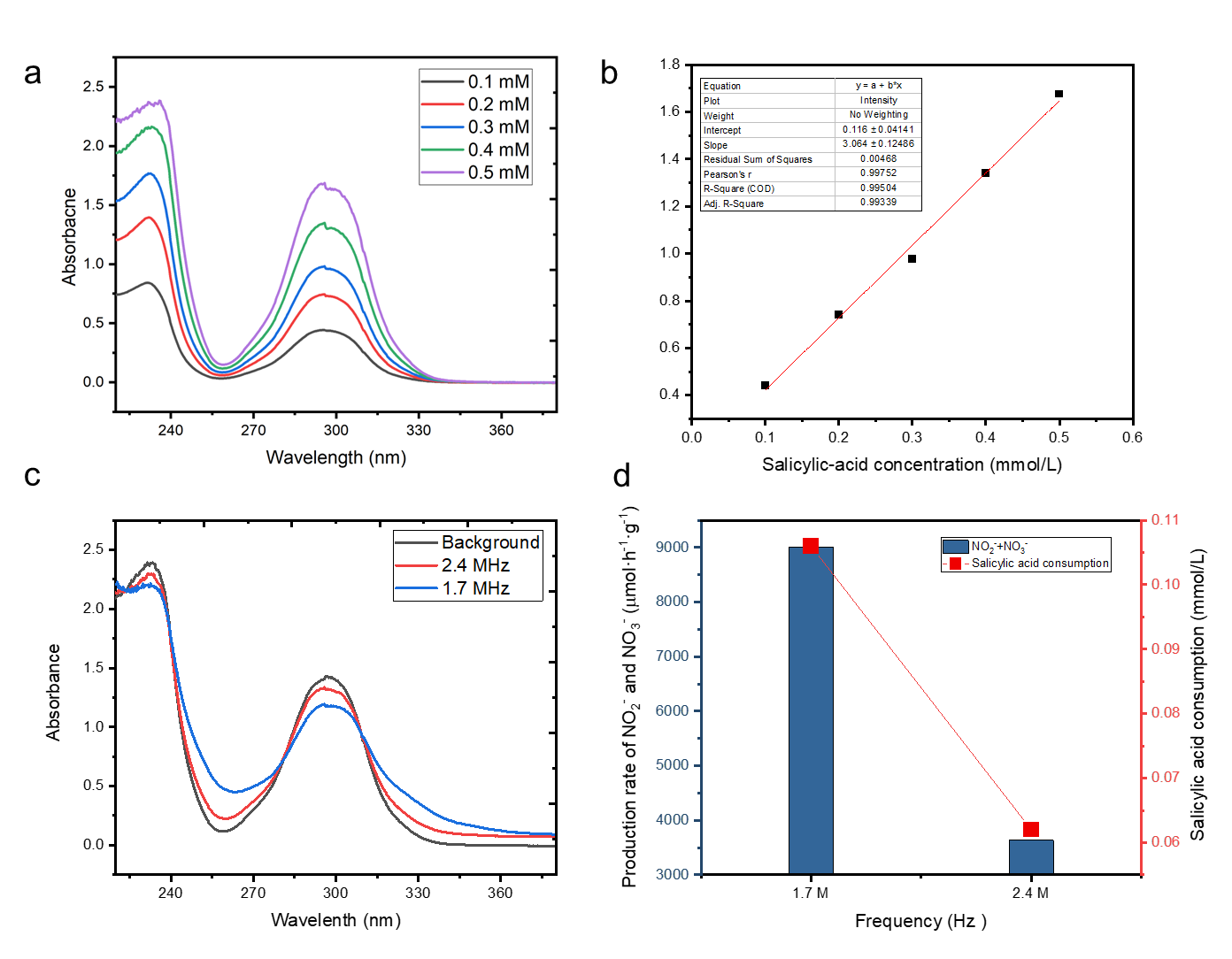
**Figure S11. Quantitative salicylic-acid probing of ·OH generation under different ultrasound conditions and its correlation with nitrogen fixation.**

**(a)** UV–vis absorption spectra of salicylic acid (SA) standard solutions (0.1–0.5 mM), used for quantifying ·OH through monitoring SA consumption at 295 nm. **(b)** Linear calibration plot of SA concentration versus absorbance at 295 nm, showing excellent linearity (R² = 0.995), enabling quantitative determination of ·OH generation. **(c)** SA-probe spectra obtained under different ultrasound frequencies (1.7 MHz and 2.4 MHz) and the background condition, illustrating the substantially higher ·OH formation at 1.7 MHz. **(d)** Direct correlation between ·OH flux (represented by SA consumption) and total nitrogen-fixation rate (NO₂⁻ + NO₃⁻). The higher ·OH production at 1.7 MHz corresponds higher NOx generation comparing with 2.4 MHz, highlighting ·OH as the primary rate-determining species.


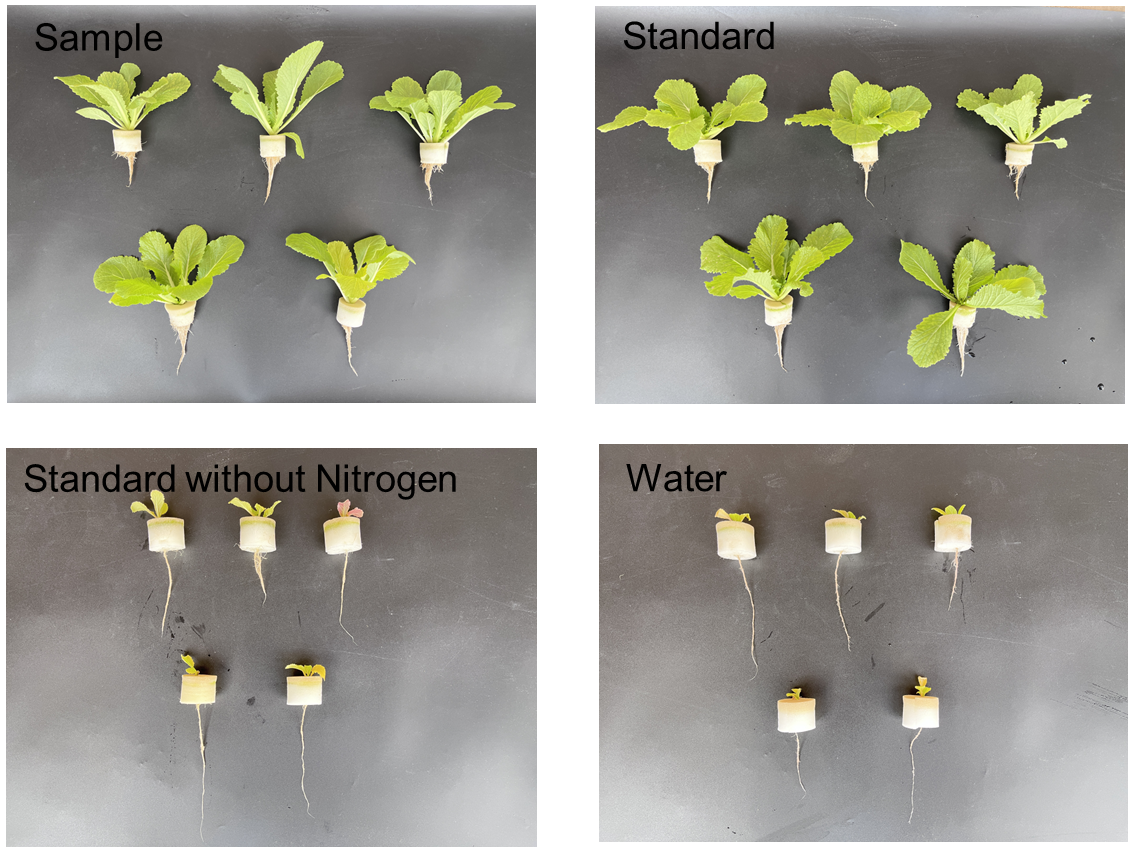
**Figure S12.** **Representative cultivation images of pakchoi under different nutrient treatments.** Photographs of plants grown for 5 weeks under Sample (HNO₃-based nutrient solution), Standard Hoagland, Standard without Nitrogen, and Water treatments, demonstrating comparable growth between Sample and Hoagland and severe suppression under N-deficient conditions.


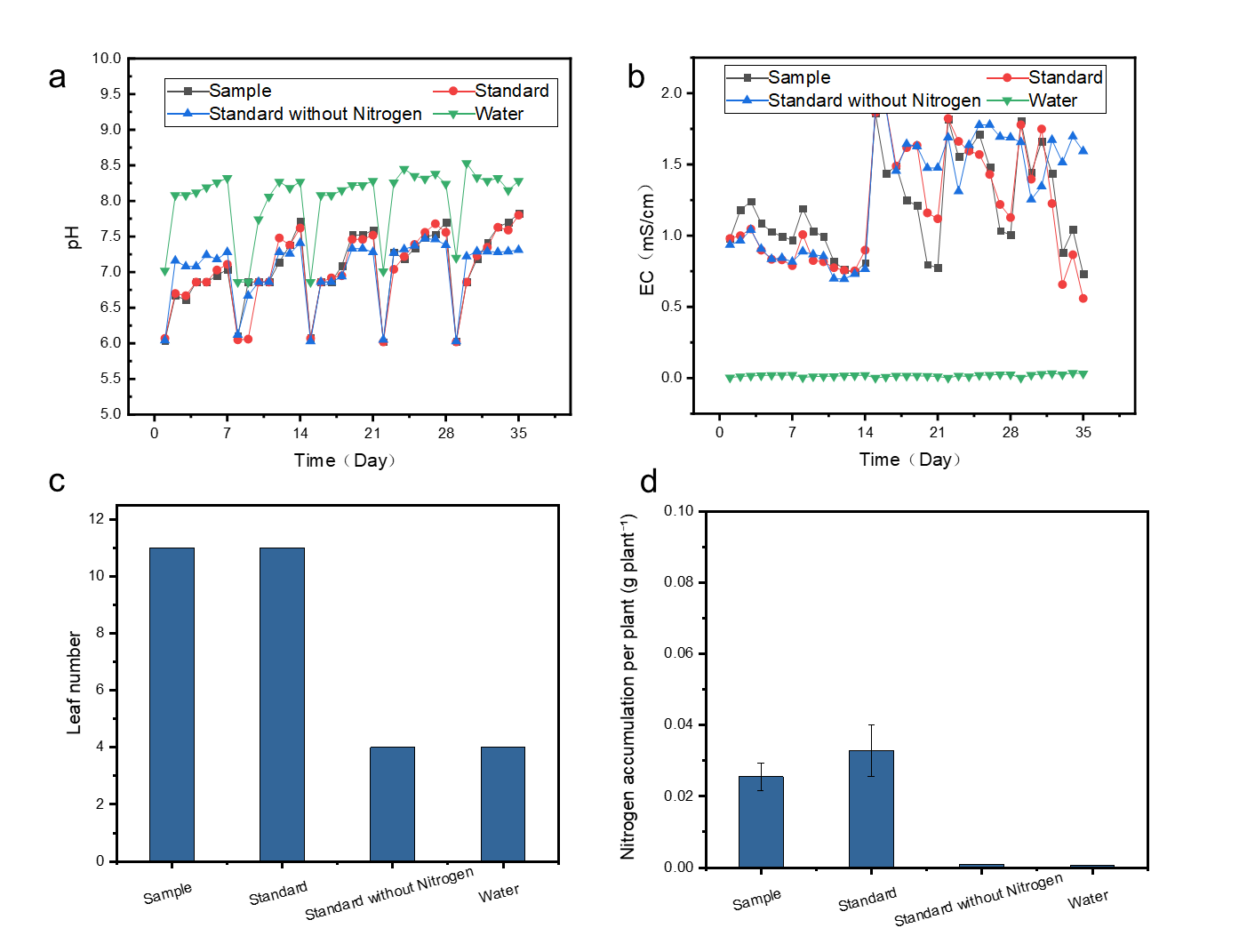


**Figure S13. Nutrient-solution stability and nitrogen uptake during pakchoi cultivation. (a)** pH variation; **(b)** EC variation; **(c)** final fresh weight; **(d)** nitrogen accumulation per plant for Sample, Standard, Standard without Nitrogen, and Water groups. Sample closely matches Hoagland control in all parameters, confirming effective fertilization performance of the produced HNO₃ solution.

These figures presents the nutrient-solution dynamics and growth performance of pakchoi under four treatments—Sample (HNO₃-based nitrogen solution produced by the ultrasound-assisted photocatalytic system), Standard (Hoagland solution), Standard without Nitrogen (N-free Hoagland), and Water—over a 5-week cultivation period. The pH profiles show that, except for the N-free and Water groups, all nutrient solutions were prepared with identical macro- and micronutrient compositions and normalized total nitrogen input, with weekly medium renewal resulting in periodic oscillations. The Sample and Standard groups exhibit nearly identical pH evolution, whereas the N-free and Water treatments display larger fluctuations due to nitrogen deficiency. Correspondingly, electrical conductivity (EC) measurements reveal similar nutrient-consumption dynamics for the Sample and Standard groups, while the N-free and Water treatments maintain consistently low EC values, reflecting the absence of nitrogen-containing nutrients. After 5 weeks of growth, the final fresh weight (mean ± SD, n ≥ 3) shows that the HNO₃-derived Sample achieves a biomass yield statistically indistinguishable from the Hoagland control, whereas N-deficient treatments suffer severe growth suppression. Total nitrogen accumulation per plant, quantified by the Kjeldahl method, likewise demonstrates comparable N uptake for the Sample and Standard groups and minimal nitrogen content in N-free and Water plants. All cultivation experiments were carried out in a daylight greenhouse at 25–35 °C, with seedlings initially raised in trays and subsequently transplanted into 100 mL black cultivation bottles at the 3–4-leaf stage.
